# Psychometric Properties of the Chinese Version of the Organization Big Five Scale

**DOI:** 10.3389/fpsyg.2021.781369

**Published:** 2021-11-16

**Authors:** Yong Meng, Boxiang Yu, Chaoping Li, Yuanmei Lan

**Affiliations:** ^1^Management Institute, Xinxiang Medical University, Xinxiang, China; ^2^School of Public Administration and Policy, Renmin University of China, Beijing, China

**Keywords:** big five personality, chinese employee, ORG-B5, scale validation, work engagement, measurement invariance

## Abstract

This study translates the Organization Big Five Scale (ORG-B5) into Chinese and tests its reliability and validity. In Study 1 (*N*=406), the ORG-B5 was translated into Chinese, and an exploratory factor analysis established the scale’s factorial validity. In Study 2 (*N*=391), confirmatory factor analyses found that the five-factor correlation model fit the data best. The results from the configural, metric, and scalar invariance models also demonstrate that the ORG-B5 is equivalent across gender, age, and work tenure. The relationship between ORG-B5 and related constructs was also explored further. This study argues that the Chinese version of ORG-B5 provides researchers with a psychometrically sound and efficient tool to assess the Big Five personality traits within organizations in the Chinese context.

## Introduction

With in-depth research being conducted on the interaction between individuals and their environment, an increasing number of researchers have begun to pay attention to the role of personality in this interaction. Over the past few decades, researchers have found a wide range of correlations between personality and life and work outcomes. For example, studies have shown that personality is related to academic success (Poropat, 2009), mortality (Jokela et al., 2013), physical and mental health (Strickhouser et al., 2017), and life satisfaction (Anglim et al., 2020). Personality has also been consistently found to influence various work outcomes such as task performance (Barrick and Mount, 1991; Judge et al., 2013), leader–member exchange (Dulebohn et al., 2012), and work engagement (Young et al., 2018). These studies on personality continue to encourage researchers in psychometrics to explore personality measurement tools more effectively.

The most well-known definition of personality is “the relatively enduring patterns of thoughts, feelings, and behaviors that reflect the tendency to respond in certain ways under certain circumstances” (Roberts, 2009, p7). The traditional approach to personality measurement focuses on characterizing people’s average behavioral tendencies, attitudes, relationships, preferences, and social skills across various situations (Costa and McCrae, 1992a). Consequently, researchers have used similar personality measurement scales irrespective of the context and have ignored the possible impact of the environment (Funder, 2006). However, some researchers have questioned the validity of this traditional approach. Judge and Zapata (2015) believe that individual personalities tend to be expressed differently according to different job-needs scenarios. Shaffer and Postlethwaite (2012) propose that personality measurement under a specific framework had better predictive validity compared to scales with no framework.

Items in personality measurement instruments with no clear situational information cause respondents to extract information from different reference frames (e.g., at work or home) to provide the corresponding context to these items (Pathki et al., 2021). Moreover, behavioral scholars have long thought that behavior is an interaction between personality traits and situations (Mischell, 1977; Mischel and Shoda, 1995; Cervone and Shoda, 1999). Noncontextualized personality measures also constitute the main difficulties and limitations that hinder the validity and application of relevant results of personality research (Robie et al., 2000; Morgeson et al., 2007). Hence, there is an apparent gap between theory and practice regarding personality measurement.

To solve these problems and restrictions, Pathki et al. (2021) integrated the frame-of-reference (FOR) theory with the knowledge-and-appraisal personality architecture (KAPA) theoretical framework to develop a new work-FOR personality measure: the 20-item Organization Big Five Scale (ORG-B5). Through a series of psychometric property tests on ORG-B5, Pathki and colleagues demonstrated that ORG-B5 is a reliable and short personality measure, more appropriate than existing measures, for organizational research.

The Big-Five personality model is a comprehensive, well-known model that describes individual personality characteristics based on five factors: conscientiousness, extroversion, agreeableness, emotional stability, and openness to experience (Costa and McCrae, 1992b). It has become the most widely adopted framework since the 1990s (John et al., 2008). Meanwhile, the complexity of personality constantly motivates researchers to develop an inventory for personality measurement with more psychometric characteristics. For example, some inventories assess personality traits using noncontextualized statements. Prominent examples include the NEO Five-Factor Inventory (Costa and McCrae, 1992b), Big Five Mini-Markers (Saucier, 1995), Big Five Inventory (John and Srivastava, 1999), IPIP Five-Factor Model scales (International Personality Item Pool, Ehrhart et al., 2008), and International Personality Item Pool-NEO Inventory (Goldberg, 1999). Other measures adopt contextualized items, while some inventories assessing the Big Five traits utilize the FOR theory: the Occupational Personality Inventory (Saville and Holdsworth Ltd, 1998), Five-Factor Model Questionnaire (Gill and Hodgkinson, 2007), and Work-Based Inventory (Bing et al., 2014).

Pathki et al. (2021) conducted a systematic review to select the best among these existing personality scales for organizational research. They screened these scales according to five criteria: content validity issues with FOR measures, language difficulties with item content, exclusive reliance on contextualization through instructions, access, and survey length. They concluded that all scales had limitations and deficiencies to varying degrees, which limited further development of personality measurement. Therefore, based on the theory of KAPA, which holds that individuals’ personalities comprise knowledge of self and environment and appraisal of self to the environment (Cervone, 2004), Pathki et al. (2021) developed the ORG-B5 as a superior alternative measure for use in organizational research.

Initially, the items of ORG-B5 were derived from the non-situational items of the classic IPIP scale (Goldberg, 1999). Pathki et al. (2021) screened and revised them according to the five aforementioned criteria. First, to avoid confusion and translation difficulties caused by varying cultural expressions (Brislin, 1980), items that had nothing to do with the work background and were difficult to understand were deleted. Second, to avoid the impact of reverse scoring on the validity and internal consistency of the scale (Chamberlain and Cummings, 1984; Colquitt et al., 2019), all reverse scoring items were also removed. Furthermore, to ensure the content validity of the measurement items, the items were chosen through the definition correspondence and definition distinctiveness methods (Anderson and Gerbing, 1991; Tracey, 1999; Colquitt et al., 2019). Third, considering that too many measurement items would lead to fatigue or response bias in the respondents (Carmines and Zeller, 1979), only four items with the highest scores in each measurement dimension were retained (Judge et al., 2003). Finally, combined with a series of empirical research results, Pathki et al. (2021) developed the 20-item ORG-B5 with five dimensions (agreeableness, conscientiousness, extraversion, openness, and emotional stability; four items in each dimension). They argued that the 20-item ORG-B5 was more beneficial for research in the workplace compared to the existing personality scales.

In China, some researchers have developed personality scales tailored to the Chinese context (Cheung et al., 1996; Wang and Cui, 2004), while others have modified the personality scales developed within the western cultural context and validated their psychometric characteristics in the Chinese context (Qian et al., 2000; Dai and Wu, 2005; Zheng et al., 2008; Yao and Liang, 2010). However, among these scales, there is no work-FOR scale developed or adapted for organizational research in China. The ORG-B5 has incremental validity in predicting workplace outcomes compared with existing personality measurement scales (Pathki et al., 2021), and has the advantage of being short, freely accessible, and based on work-FOR. Therefore, this study bridges the research gap by applying and validating the ORG-B5 in the Chinese organizational context by developing a Chinese version of the ORG-B5.

This study translates the ORG-B5 into Chinese and tests its reliability and validity with a sample of Chinese employees. To accomplish this goal, we gathered data from full-time employees in China through online surveys. In Study 1, we translated the ORG-B5 and conducted an exploratory factor analysis (EFA) for its factor structure. In Study 2, we collected data from a new sample and future tested the validity of the ORG-B5 in this Chinese sample.

## Study 1

Study 1 was conducted with two primary goals. The first was to translate the ORG-B5 into the Chinese language. The second was to test the number of core factors in the ORG-B5 by examining its factor loading patterns.

### Scale Translation

We used the classic back-translation method (Brislin, 1980) to ensure accuracy. The scale translation process consisted of five steps. In step 1, all items were translated into Chinese by the third and fourth authors, who are bilingual (Chinese and English) experts in organizational psychology. In step 2, we jointly compared and evaluated the translations and reached a consensus on the translation. In step 3, the translated Chinese version was then back translated by another bilingual academic researcher who was not otherwise related to this study. In step 4, after back-translation, we invited two bilingual organizational psychologists to compare the back-translated versions with the original English items regarding the meaning of the items. Finally, we adjusted the wording of some items. For example, in item 4 of the agreeableness dimension, the original was expressed as: I care about others at work. Considering that the modest and introverted culture may influence the employees in the Chinese context (Zhu et al., 2015), we highlight the employees’ initiative in item translation, and the adjusted expressions are as follows: At work, I take the initiative to care about my colleagues around me. After discussion, we concluded that the Chinese version of the adjusted ORG-B5 is consistent with the original ORG-B5.

### Participants

The sample consisted of 406 full-time employees in China. Of the participants, 37 (9.1%) were female and 369 (90.9%) were male, with a mean age of 32.9years (*SD*=8.08; range=21.0–61.0years). Education levels consisted of high school education (*n*=69, 17.5%), undergraduate degree (*n*=310, 76.4%), and master’s degree or above (*n*=27, 6.6%). Two participants (0.5%) did not respond to this question. The average work tenure was 11.3years, with an average of 10.2years in the current job. Most participants were front-line employees (*n*=215, 53.0%) and intermediate professionals (*n*=131, 32.3%), while others were middle and senior managers (*n*=60, 14.8%).

### Procedure

In study 1, we adopted convenience sampling. We formed our first batch of participants by contacting—over social media—about 30 alumni and colleagues from a university in central China. After the first group of participants completed the research project, they were asked to release recruitment information in the organization they worked for and recruit full-time staff to participate in our research. Confidentiality and anonymity were assured.

A link to an online survey was sent to the personal email address of the participants. First, an online informed consent form was presented on the first page. Next, participants entered the formal research interface of this study, which contained the 20-item Chinese version of the ORG-B5, and questions regarding demographic information such as age, gender, work tenure, and job position.

As the questionnaire survey was conducted online, to ensure effective data collection, we used specific methods to screen the online data of the original 498 participants (Porter et al., 2019). First, based on multivariate outlier analysis, 55 outliers who responded carelessly or who did not appear to have made a sincere effort were eliminated based on the Mahalanobis distance. Second, through the long string screen analysis, we eliminated participants (*n*=37) who had the same response to all questions. The final sample included 406 participants. These steps were implemented through the R package “careless” (Yentes and Wilhelm, 2018).

### Results

The development of the ORG-B5 scale (Pathki et al., 2021) followed the five-dimension hypothesis of the Big-Five personality theory. The ORG-B5 scale was composed of five factors related to each other. Therefore, we expected that the Chinese version of the ORG-B5 scale would also consist of five interrelated factors. Simultaneously, considering that the distribution of variables does not necessarily conform to multivariate normality, we carried out an EFA using the principal axis factoring method with a Promax rotation in Jamovi Version 1.6.5 (Costello and Osborne, 2005; Kahn, 2006). The Kaiser–Meyer–Olkin measure of sample adequacy was 0.916, while Bartlett’s test of sphericity was significant (*p*<0.001), indicating that the samples collected in this study met the premise of factor analysis.

In addition, we tested the factor structure using parallel analysis (Horn, 1965), one of the most accurate methods for determining the number of factors that should be extracted (Hayton et al., 2004). It randomly generates a simulation data matrix (the number of variables and observations are the same as the actual data) and then compares the eigenvalues of the simulation data with the actual data. Eigenvalues in the sample data that are more prominent than the simulation data should be retained (O’connor, 2012; Duffy et al., 2017).

Combined with the results of the scree plot, parallel analysis, and percentage of variance explained by the factors, we argued that the Chinese version of the ORG-B5 is consistent with the original ORG-B5 measure and contains five different factors: the five dimensions of personality (agreeableness, conscientiousness, extraversion, openness, and emotional stability). The factor load of each item is shown in Table 1. Except for item 12 (“I talk a lot at work”), all items were clustered on their respective primary factors, with factor loadings above 0.35 (range of 0.39–0.90).

**Table 1:** Results of the EFA on the Chinese version of the ORG-B5 in Study 1.

	Factor Loading				
Items	F1	F2	F3	F4	F5
**Agreeableness**
Q1	0.03	0.05	**0.56**	0.02	0.09
Q2	0.03	−0.02	**0.87**	0.04	−0.03
Q3	0.02	0.14	**0.55**	−0.16	0.12
Q4	0.08	0.06	**0.70**	0.03	0.05
**Conscientiousness**					
Q5	**0.59**	0.08	0.19	0.01	0.01
Q6	**0.90**	0	0	0.04	−0.03
Q7	**0.91**	−0.03	−0.02	−0.03	−0.03
Q8	**0.73**	0.07	0.01	0.01	0.14
**Extraversion**					
Q9	0.06	−0.19	0.24	0.15	**0.54**
Q10	0.02	0	0.02	0	**0.78**
Q11	0.01	0.2	−0.02	0.05	**0.71**
Q12	0.02	**0.51**	−0.08	−0.01	**0.33**
**Openness**					
Q13	0.05	**0.39**	0.13	0.08	0.21
Q14	0.05	**0.84**	0.01	0.01	0
Q15	0.04	**0.69**	0.08	0.05	0.04
Q16	−0.07	**0.64**	0.02	0.11	0.03
**Emotional stability**					
Q17	−0.07	0.13	0.04	**0.58**	0.08
Q18	0.01	0.07	−0.15	**0.69**	0.05
Q19	0.01	0.05	0.16	**0.69**	−0.12
Q20	0.12	−0.04	0	**0.68**	0.12

*N*=406. Bolded corresponding to the factor they load on.

Item 12 had a factor loading of 0.51 and 0.33 on factors 2 (Openness) and 5 (Extraversion). In the study by Pathki et al. (2021), item 12 belonged to the Extraversion dimension. However, according to Study 1, item 12 belonged to both the Openness and Extraversion dimensions. The loading on the Openness factor was greater than that on Extraversion, which indicates an inconsistency with the original scale. This difference may be because of the unique understanding of this item by participants in the Chinese cultural context. However, to ensure the measurement validity of subsequent studies, we decided to delete item 12.

Table 1 shows that the Chinese version of the ORG-B5 is composed of five different factors: Agreeableness (explaining 12.5% of the variance in the ORG-B5), Conscientiousness (explaining 14.8% of the variance), Extraversion (explaining 10.5% of the variance), Openness (explaining 11.7% of the variance), and Emotional stability (explaining 11.0% of the variance). The five factors explain 60.4% of the total variance of the 19 items. Furthermore, we tested the internal consistency coefficients of ORG-B5 total and of all five subscales: 0.91 (total), 0.82 (agreeableness), 0.90 (conscientiousness), 0.81 (extraversion), 0.82 (openness), and 0.80 (emotional stability). Finally, we tested the correlations among the subscales and found that the five factors of the ORG-B5 were significantly correlated with each other (range of 0.32–0.73).

## Study 2

In study 2, we examined the factor structure and model fit of the Chinese version of the ORG-B5 using a series of confirmatory factor analyses (CFAs), including a correlated five-factor model, a single factor model, and a second-order five-factor model. Furthermore, we used the multigroup confirmatory analysis to test the measurement invariance of the scale across the gender, age, and work tenure groups in China. Finally, to verify the criterion-related validity of the ORG-B5 scale, we combined the development study of the initial scale (Pathki et al., 2021) and the results of related studies (Robins et al., 2001; González Gutiérrez et al., 2005; Jones et al., 2011; Hosie et al., 2014). We hypothesized a significant positive correlation between each trait dimension of the ORG-B5 and job engagement but a significant negative correlation with counterproductive workplace behavior (CWB). We selected aggressive behavior, self-esteem, and work well-being (WWB) as criteria to further verify the validity of ORG-B5 in personality measurement.

After referring to the development and research of ORG-B5 and the existing research on personality, we predicted that each dimension of ORG-B5 will have a significant positive correlation with work engagement and a significant negative correlation with CWB (Pathki et al., 2021). For subjective well-being, researchers have confirmed that each dimension of the Big-Five personalities has a significant relationship with subjective well-being (González Gutiérrez et al., 2005). Considering that ORG-B5 is designed for organizational situations, we selected the work well-being subscale of the subjective well-being scale as the criterion and predicted that it has a significant positive correlation with all dimensions of the ORG-B5. For self-esteem (Robins et al., 2001), we verified the significant relationship between self-esteem and all dimensions of personality. We believe that this relationship will remain stable in the workplace; therefore, we predicted that individuals who maintain high scores in all dimensions of the ORG-B5 will also have a higher level of self-esteem. For aggressiveness, the current research results show a significant negative relationship between conscientiousness, emotional stability, and agreeableness, while extroversion and openness are not significantly related (Jones et al., 2011; Hosie et al., 2014). Considering that the design of the ORG-B5 is based on work-FOR, and Pathki et al. (2021) have confirmed that it has better measurement validity than non-FOR scales, we predict that all dimensions of the ORG-B5 will show a significant negative correlation with aggressiveness.

### Participants and Procedure

The recruitment procedure of Study 2 was similar to Study 1. The survey link in Study 2 contained the survey information page, the 19-item Chinese version of the ORG-B5 from Study 1, the scales of other constructs used in Study 2, and questions regarding demographic information such as age, gender, education background, tenure, and job position.

A total of 474 full-time employees were originally recruited. However, following the same procedure used in Study 1, a total of 83 participants were excluded from analysis: 71 participants were outliers based on multivariate outlier analysis and 12 failed to pass the long string screen test (the number of consecutive identical responses to different items exceeded half of the total times). This was implemented through the R package “careless” (Yentes and Wilhelm, 2018).

The final sample in study 2 included 391 adult employees from various jobs. Of the participants, 142 (36.3%) were female and 249 were male (63.7%), with an average age of 38.9years (*SD*=8.64, range=21–59years). Participants’ education was categorized into high school education (*n*=46, 11.8%), undergraduate degree (*n*=268, 65.6%), or master’s degree and above (*n*=76, 19.4%). One participant (0.3%) did not respond to this question. The average working tenure was 16.7 (*SD*=9.47) years, with an average of 13.4 (*SD*=8.68) years in the current position. Most participants were front-line employees (*n*=147, 37.6%), followed by middle-level (*n*=112, 28.6%) and senior professionals (*n*=40, 10.2%). Other participants worked as grass-roots managers (*n*=31, 7.9%), middle managers (*n*=55, 14.1%), and senior managers (*n*=6, 1.5%).

### Measures

#### Personality Trait

The 19-item Chinese version of the ORG-B5 validated in Study 1 was used to assess personality traits. The internal consistency of the ORG-B5 in this study was 0.92. The five dimensions of the ORG-B5 had acceptable internal consistency coefficients (Cronbach’s alpha) as follows: 0.80 (agreeableness), 0.88 (conscientiousness), 0.83 (extraversion), 0.81 (openness), and 0.83 (emotional stability).

Considering that some researchers have proposed that McDonald’s omega (ω) has more advantages than Cronbach’s alpha in indicating reliability (Cortina et al., 2020), we used omega as the supplementary index of reliability in Study 2 *via* Jamovi (Hayes and Coutts, 2020). The omega values of ORG-B5 were 0.80 (agreeableness), 0.88 (conscientiousness), 0.84 (extraversion), 0.82 (openness), and 0.84 (emotional stability).

#### Work Engagement

The Utrecht Work Engagement Scale (UWES3) ultra-short version was used to assess work engagement (Schaufeli et al., 2019). The Chinese version comes from the official website of the scale. Three items were rated on a 5-point Likert scale, ranging from 1 (never) to 5 (always). The scale includes three dimensions: vigor (At my work, I feel bursting with energy), dedication (I am enthusiastic about my job), and absorption (I am immersed in my work). Schaufeli et al. (2019) have confirmed that the UWES3 and UWES9 have similar psychometric characteristics. We selected the ultra-short version of the UBW scale as it accords with the study of Pathki et al. (2021), who state that long measures are less pragmatic in organizational research, and a short measure is desirable. In this study, the reliability of the total score was 0.85 (α) and 0.86 (ω).

#### Counterproductive Workplace Behavior

We measured CWB using the scale developed by Dalal et al. (2009). Bai et al. (2016) provided reasonable reliability estimates of this scale—higher than 0.80—based on two Chinese samples. In this study, eight items were rated using a 5-point scale (1=strongly disagree, 5=strongly agree). Participants were asked to indicate their agreement with statements such as “In my work, I do something that has nothing to do with my work.” The reliability coefficient in our study was 0.89 (α) and 0.91 (ω).

#### Work Well-Being

This factor was measured using the work well-being subscale of the employee well-being scale developed by Zheng et al. (2015). The subscale consists of six items rated on a 5-point Likert scale, ranging from 1 (strongly disagree) to 5 (strongly agree). Examples include the following: “I am basically satisfied with the specific content of my work.” In this study, the reliability of the work well-being scale was 0.90 (α) and 0.91 (ω).

#### Self-Esteem

This study used the Self-Esteem Scale (SES) developed by Rosenberg (1965) to measure whether individuals hold a positive or negative evaluation about themselves. Participants responded to 10 questions on a 5-point Likert scale (1=very inconsistent, 5=very consistent). Items included statements such as “I feel like a valuable person, at least at the same level as others.” A study by Yang et al. (2021) showed that the SES scale was reliable in Chinese samples. The reliability coefficient of SES in our sample was 0.88 (α) and 0.89 (ω).

#### Aggression

This factor was measured using the short form of the Aggression Questionnaire (Bryant and Smith, 2001). The Chinese version of this scale was revised by Li et al. (2011), and it has been proved to have good reliability (0.6–0.89) among Chinese samples. We selected six items from the two subscales of physical aggression and verbal aggression to measure the aggressive behavior of the participants. Participants were asked to judge the descriptions presented according to their actual situations (e.g., “If someone hits me, I will fight back.”), which were rated on a 5-point scale (1=strongly disagree, 5=strongly agree). Cronbach’s alpha coefficient for this scale in our study was 0.73. The omega coefficient has the same value.

### Results

#### Confirmatory Factor Analyses

To test the factor structure of the 19-item Chinese version of the ORG-B5, we used the “lavaan” package (Rosseel, 2012) in R 4.10 with robust maximum likelihood estimation. In Study 2, we used three separate models to assess the factor structure: a five-factor correlational model, a unidimensional one-factor model, and a higher order model. Table 2 shows the goodness-of-fit indices related to these models.

**Table 2:** Confirmatory Factor Analyses in Study 2.

Model	*χ* ^2^ *(df)*	CFI	RMSEA	90%CI	SRMR	AIC	BIC	TLI
Correlated Five-Factor Model	356.77 (142)	0.944	0.062	0.05, 0.07	0.054	14,039	14,230	0.933
Single Factor Model	1368.13 (152)	0.684	0.143	0.14, 0.15	0.101	15,030	15,181	0.644
Second Order Five Factor Model	408.62 (171)	0.932	0.067	0.06, 0.08	0.066	14,081	14,252	0.921

*N*=391. *χ*^2^=chi-square statistic; CFI, comparative fit index, RMSEA, root mean square error of approximation, 90% CI refers to 90% confidence intervals for the RMSEA values, SRMR, standardized root means square residual, AIC, Akaike Information Criterion, BIC, Bayesian Information Criterion, TLI, Tucker–Lewis Index.

**Figure 1:**
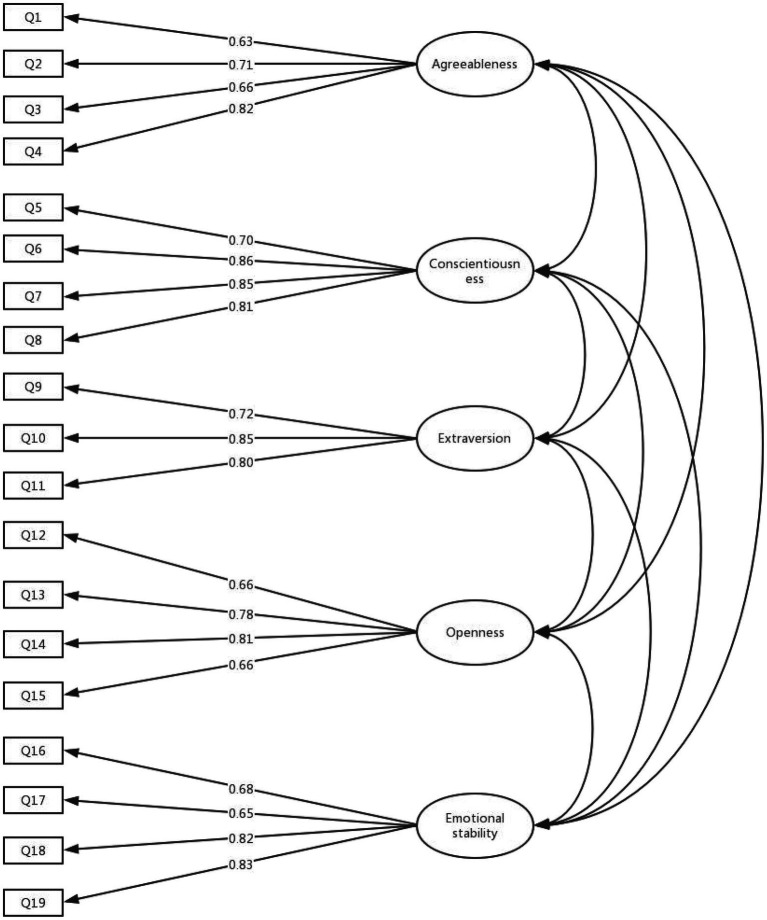
Confirmatory factor analysis result of the five-factor correlational model in Study 2.

The correlated five-factor model consisted of five independent dimensions of personality. Each item carried a load on its dimension and allowed the five dimensions to be related (Figure 1). Similar to the scale development research, this model had an acceptable fit to the data, *χ*^2^ (142)=356.772, *p*<0.001, TLI=0.933, CFI=0.944, RMSEA=0.062, 90% CI [0.05, 0.07], and SRMR=0.054. All items were significantly loaded on the primary factor (load ranges from 0.63 to 0.86). The good fitting of the correlated five-factor model indicated that the five factors of the ORG-B5 were independent of each other to a great extent.

By contrast, the unidimensional one-factor model allowed all items to be loaded on a single factor. Structural equation modeling (SEM) fitting results showed that this model had poor fit to the data: *χ*^2^ (152)=1368.125, *p*<0.001, TLI=0.644, CFI=0.684, RMSEA=0.143, 90% CI [0.14, 0.15], and SRMR=0.101. The CFI, RMSEA and SRMR values of this model could not meet the acceptable standard (CFI≥0.90, RMSEA and SRMR ≤0.10). Furthermore, when this model was compared with the correlated five-factor model, ∆*χ*^2^ (10)=1011.353, *p*<0.001, and the CFI and RMSEA change was much greater than 0.01 (∆CFI=0.26, ∆RMSEA=0.081), indicating that this change between models was significant and the models were practically different (Cheung and Rensvold, 2002).

The correlated five-factor model was also compared with the second-order five-factor model, which required each of the five latent personality dimensions to regress onto a higher order overall personality factor rather than being correlated. This model had a good fit to the data: *χ*^2^ (171)=408.622, *p*<0.001, TLI=0.921, CFI=0.932, RMSEA=0.067, 90% CI [0.06, 0.08], and SRMR=0.066. Upon comparison of the two models, ∆*χ*^2^ (29)=51.85, *p*<0.01, ∆CFI=0.012, and ∆RMSEA=0.005; the change in CFI and RMSEA was greater than 0.01. Therefore, although the fitting indexes of the second-order five-factor model performed well, the correlated five-factor model fit better, and practical differences exist between the two models. Meanwhile, the comparison of AIC and BIC values between different models also shows that the related five-factor model has the minimum AIC and BIC values and fit the data best.

#### Factorial Invariance

We tested the invariance of the correlated model in terms of sex, age, and work tenure. Regarding the integrative paradigm of measurement invariance test proposed by Vandenberg and Lance (2000), we first divided the data into two categories according to these variables. For gender, we compared males versus females. For the age group, we referred to previous studies (Duffy et al., 2017) to split the group at the mean (38.9) to create two categorical groups. For work tenure, according to the average working life (13.4) of the sample, the sample was divided into the relatively long working tenure group and short working tenure group.

After grouping the data, we tested the model’s configural, metric, and scalar invariance by controlling the model structure, factor loadings, and indicator intercepts of the model in turn. The establishment of each equivalent model was based on the previous model. Table 3 shows the goodness-of-fit indices related to these models.

**Table 3:** Test of Measurement Invariance Across Gender, Age, Work tenure in Study 2.

Model	*χ* ^2^ *(df)*	TLI	CFI	RMSEA [90%CI]	SRMR	∆CFI	∆RMSEA	∆TLI
**Gender**								
M0 (configural)	566.93 (284)	0.913	0.928	0.071 [0.063, 0.080]	0.059			
M1 (metric)	583.36 (298)	0.917	0.927	0.070 [0.062, 0.078]	0.064	0.001	0.001	0.004
M2 (scalar)	623.36 (312)	0.913	0.921	0.071 [0.063, 0.080]	0.066	0.006	0.001	0.004
**Age**								
M0 (configural)	516.51 (284)	0.928	0.94	0.065 [0.056, 0.074]	0.058			
M1 (metric)	533.52 (298)	0.931	0.94	0.064 [0.055, 0.072]	0.064	0	0.001	0.003
M2 (scalar)	549.7 (312)	0.933	0.939	0.062 [0.054, 0.071]	0.064	0.001	0.002	0.002
**Work tenure**								
M0 (configural)	539.72 (284)	0.922	0.935	0.068 [0.059, 0.077]	0.061			
M1 (metric)	556.41 (298)	0.925	0.934	0.067 [0.058, 0.075]	0.063	0.001	0.003	0.003
M2 (scalar)	566.17 (312)	0.929	0.935	0.065 [0.056, 0.073]	0.064	0.001	0.002	0.004

For gender, the configural model had an acceptable fit index, *χ*^2^ (284)=566.93, *p*<0.001, TLI=0.913, CFI=0.928, RMSEA=0.071, 90% CI [0.06, 0.08], and SRMR=0.059. Fit was similar for the metric model, *χ*^2^ (298)=583.36, *p*<0.001, TLI=0.917, CFI=0.927, RMSEA=0.070, 90% CI [0.06, 0.08], SRMR=0.064, and the configural and metric models did not significantly differ (∆TFI=0.004, ∆CFI=0.001, ∆RMSEA=0.001). A change of the index less than 0.01 indicates that there is no substantial difference between the two models (Cheung and Rensvold, 2002). Fit was also similar for the scalar model, *χ*^2^ (312)=623.36, *p*<0.001, TLI=0.913, CFI=0.921, RMSEA =0.071, 90% CI [0.06, 0.08], SRMR=0.066. The scalar model was not significantly different from the metric model (∆TFI=0.004, ∆CFI=0.006, ∆RMSEA=0.001). Therefore, factor structure and indicator intercepts were maintained across the male and female groups. Following the same model comparison procedure, the factorial invariance of ORG-B5 was also supported in different groups of age and work tenure (Table 3). Therefore, for the Chinese version of the ORG-B5, factor structure and indicator intercepts were maintained across gender, age, and work tenure.

#### Criterion-Related Validity of the Chinese Version of the ORG-B5

In the last part of Study 2, we tested whether the Chinese version of ORG-B5 was significantly associated with CWB, engagement, work well-being, self-esteem, and aggressiveness to prove the criterion-related validity of this scale. In addition, we also explored the relations between the scale and some demographic variables. Table 4 presents the means, standard deviations, and correlations of variables.

**Table 4:** Descriptive Statistics and Correlations in Study 2.

Variable	1	2	3	4	5	6	7	8	9	10	11	12	13
1. Age	–												
2. Gender	0.28^***^	–											
3. Work tenure	0.77^***^	0.11^*^	–										
4. Aggreeableness	0.04	−0.03	0.06	(0.80)									
5. Conscientiousness	0.10	0	0.09	0.65^***^	(0.88)								
6. Extraversion	−0.04	0.13^*^	−0.02	0.55^***^	0.50^***^	(0.83)							
7. Openness	0.05	0.21^***^	0.01	0.51^***^	0.46^***^	0.63^***^	(0.81)						
8. Emotional stability	0.01	0.15^**^	−0.05	0.40^***^	0.32^***^	0.47^***^	0.54^***^	(0.83)					
9. CWB	0.10^*^	0.11^*^	0.07	−0.33^***^	−0.38^***^	−0.35^***^	−0.27^***^	−0.29^***^	(0.89)				
10. WWB	0.03	0	0.07	0.42^***^	0.42^***^	0.45^***^	0.47^***^	0.42^***^	−0.40^***^	(0.90)			
11. Engagement	0.04	−0.03	0.08	0.43^***^	0.43^***^	0.40^***^	0.46^***^	0.34^***^	−0.41^***^	0.61^***^	(0.85)		
12. Self-esteem	0.12^*^	−0.02	0.12^*^	0.42^***^	0.47^***^	0.32^***^	0.33^***^	0.29^***^	−0.34^***^	0.44^***^	0.44^***^	(0.88)	
13. Aggressiveness	0.10^*^	0.11^*^	0.08	−0.17^***^	−0.20^***^	−0.18^***^	−0.09	−0.25^***^	0.46^***^	−0.24^***^	−0.15^**^	−0.29^***^	(0.73)
*M*	38.9	—	13.4	4.21	4.53	3.98	3.88	3.65	1.66	3.93	3.87	4.07	2.44
*SD*	8.64	—	8.92	0.57	0.56	0.74	0.66	0.79	0.59	0.60	0.63	0.57	0.68

*N*=391. CWB, counterproductive workplace behavior; WWB, work well-being. Cronbach’s α values appear along the diagonal in parentheses.

* *p*<0.05;

** *p*<0.01;

*** *p*<0.001.

Consistent with our expectations, the results presented in Table 4 show that all five dimensions of the ORG-B5 are significantly positively correlated with work engagement (*r*=0.34 to 0.52), WWB (*r*=0.42 to 0.56), and self-esteem (*r*=0.29 to 0.46) and negatively related to CWB (*r*=−0.29 to −0.41). To further verify the relationship between ORG-B5 and workplace outcome variables, we conducted a series of regression analysis of each dimension of ORG-B5 with engagement and CWB (age, gender, and work tenure as control variables). The results of regression analysis show that each dimension of ORG-B5 has a significant predictive effect on engagement: agreeableness (*b*=0.47, *SE*=0.05, *p*<0.001), conscientiousness (*b*=0.48, *SE*=0.05, *p*<0.001), extraversion (*b*=0.35, *SE*=0.04, *p*<0.001), openness (*b*=0.47, *SE*=0.04, *p*<0.001), and emotional stability (*b*=0.28, *SE*=0.04, *p*<0.001). This result was repeated in CWB: agreeableness (*b*=−0.34, *SE*=0.05, *p*<0.001), conscientiousness (*b*=−0.41, *SE*=0.05, *p*<0.001), extraversion (*b*=−0.29, *SE*=0.04, *p*<0.001), openness (*b*=−0.28, *SE*=0.04, *p*<0.001), and emotional stability (*b*=−0.23, *SE*=0.04, *p*<0.001). The criterion relation validity of ORG-B5 was further supported. However, for aggressiveness, except for the openness dimension, all the other personality dimensions demonstrated a significant negative correlation (*r*=−0.17 to −0.25), while the correlation between openness scores and aggressiveness scores were not significant (*r*=−0.09, *p*>0.05). Notably, all significant variable relationships are presented at the level of 0.001, which provides some support that the measure with FOR theory will have more measurement validity (Table 4).

Correlations between the ORG-B5 and demographic variables revealed no significant relationship between personality and its dimensions and age and work tenure. However, there was a significant correlation between gender and extroversion, openness, and emotional stability. The result shows that, at work, males may have more extroverted, open-minded, and emotionally stable personality traits compared to females.

## General Discussion

This study aimed to translate the ORG-B5 into Chinese and then demonstrate its reliability and validity in the Chinese organizational context. To this end, two studies involving a combined total of 797 participants demonstrated that the Chinese version of the ORG-B5 could be an effective instrument for evaluating the big five personality traits of individuals in work situations. In Study 1, we translated ORG-B5 into the Chinese language and carried out EFA to explore the rationality of its factor structure. The results support the construction of five factors and are consistent with the original ORG-B5. Subsequently, we collected data from a new sample to confirm the factor structure of the Chinese version of ORG-B5 using SEM. In addition, we tested the measurement invariance of the ORG-B5 across gender, age, and work tenure samples and examined the relationship between different personality dimensions and related constructs to verify its validity. Although the measurement items are slightly different from the initial ORG-B5, these studies reveal that the Chinese version of the ORG-B5 is a reliable and valid measure of personality traits in the context of Chinese organizations.

To explore whether the factor structure of the Chinese version of the ORG-B5 is consistent with that of the original ORG-B5, we first carried out EFA. The EFA results show that except for item 12 (I talk a lot at work), all projects evidenced adequate factor loadings on their primary factors and low cross-loading on other dimensions. Item 12 had a factor load in both the openness and extroversion dimensions of the B5. It indicated a higher factor load in the openness dimension of the Chinese version of the scale compared to the extroversion dimension to which it was assigned to in the original ORG-B5 (Pathki et al., 2021). Through specific analysis of item 12, we believe that there is a certain degree of overlap between the content of “I talk a lot at work” and the connotation of item 13, “At work, I enjoy hearing different ideas.” From personality characteristics, extroversion focuses on interpersonal interaction ability and emphasizes individual sociality and initiative. Openness focuses on individuals’ attitude toward knowledge and emphasizes that individuals explore things and seek understanding (John et al., 2008). In the Chinese cultural context, both “talk” and “hear” encompass the individual’s attitude toward knowledge. Compared with the interpersonal interaction attribute of “talk,” it is more likely that participants focus on exchanging their ideas with colleagues to gain a different understanding in the workplace. Therefore, item 12 had a load in both the openness and extroversion dimensions, with a higher load in the openness dimension. Finally, through group discussion and comprehensive consideration, we deleted item 12 in the final version and retained the remaining three items of the extroversion dimension. Overall, the EFA conducted in Study 1 suggested that the Chinese version of the ORG-B5 consists of five factors representing distinct subscales. Accordingly, we further carried out the CFA for this scale.

To confirm the factor structure of the Chinese version of the ORG-B5 obtained from Study 1, we conducted a series of CFAs. We examined three separate models in Study 2: a correlational five-factor model, a single-factor model, and a second-order five-factor model. In the development study of the original ORG-B5, Pathki et al. (2021) took the five-factor correlation model as the best fit, and this optimal factor structure has been further verified. To verify the superiority of the related five-factor model, we also designed the single-factor model and the second-order five-factor model for comparison. The analysis results of the fitting index of each model show that the correlation model has the best fitting effect for the sample data compared with other models. The result further shows that the ORG-B5 scale has good structural validity. The five personality dimensions measured by this scale are independent of each other to a great extent, and each dimension has its unique connotation. At the same time, these five factors remain related to each other and together constitute the overall personality of the individual.

In addition, to verify the invariance of the Chinese version of the ORG-B5 scale structure across gender, age, and work tenure, we tested for measurement invariance. According to the test paradigm of measurement invariance, configural, metric, and scalar models of different groups were tested and compared successively (Vandenberg and Lance, 2000). The sequential comparison of different restriction models showed no significant difference, and the changes of the fitting indexes were less than the threshold value judged by difference (Cheung and Rensvold, 2002; Nye and Drasgow, 2011). These suggest that the factor structure, factor loadings, and indicator intercepts were maintained across gender, age, and work tenure, which can be interpreted to imply that the scale’s construct has the same meaning in both subsamples. Thus, measurement invariance was supported.

Previous studies on the relationship between individual personality characteristics and workplace outcome variables have shown that each dimension of the Big Five has a positive association with work engagement (Young et al., 2018; Pathki et al., 2021), and a negative association with CWB (Kluemper et al., 2015). Work engagement involves a high level of vigor, absorption, and dedication (Schaufeli et al., 2002). The results of this study validate and support these hypotheses. First, individuals who showed extroversion in their personality were more likely to be energetic at work, and the high level of interpersonal involvement and vitality enabled them to stay energetic. Second, individuals with high scores of openness and emotional stability were more absorbed at work. Openness can cause people to love exploring and speculating about the world, while emotional stability can help individuals stay calm when dealing with emergencies at work and be less affected by negative emotions. Finally, the individual’s dedication at work was closely related to the conscientiousness and agreeableness traits of personality. Individuals with high agreeableness were tolerant, trusting, and compassionate toward their colleagues at work. Furthermore, conscientiousness can enable individuals to assume a reliable role at work and their higher goal orientation and responsibility orientation makes them more willing to contribute.

Furthermore, we examined the relationship between each dimension of the ORG-B5 and non-workplace outcome variables such as subjective well-being, self-esteem, and aggressiveness, to explore whether there is a commonality between personality measurement under the work-FOR and traditional personality scales. The test results partially support the hypothesis. For self-esteem and WWB, the test results are consistent with existing studies (Robins et al., 2001; González Gutiérrez et al., 2005). Each dimension of the ORG-B5 had a significant positive relationship with self-esteem and WWB. We predicted that every dimension of ORG-B5 would negatively affect aggressiveness; however, the relationship between openness and aggressiveness was not significant.

A previous meta-analysis found that agreeableness, conscientiousness, and emotional stability were good predictors of aggressiveness across the five dimensions of the Big Five; however, extraversion, openness, and aggressiveness were not significantly related (Jones et al., 2011). Given that these findings are based on noncontextualized personality measures, this may limit the discovery of authentic relationships between variables to some extent (Pathki et al., 2021). We argue that the personality traits of openness and extroversion can make individuals more proactive in communicating with colleagues, superiors, and partners and maintain a more inclusive and open attitude in the process. Therefore, we predict that not only do the ORG-B5 dimensions of emotional stability, conscientiousness, and agreeableness conform with the existing literature, but the dimensions of openness and extroversion are related to aggressiveness as well. However, the results of the correlation analysis only supported our prediction of the extroversion dimension, and the relationship between openness and aggressiveness was not significant in the context of work.

The relationship between extraversion and aggressiveness makes sense because at the workplace, interpersonal activity is generally limited by a specific hierarchy, out of respect for the superior, or subordinate care. The same sense of cooperation requires employees to show as little aggression as possible, for a lasting and stable relationship. However, the insignificance of the relationship between openness and aggressiveness is perhaps because openness tends to be associated with a cognitive style and is less associated with behaviors and attitudes exhibited by individuals. In addition, the significant relationship between extraversion and aggressiveness also suggests that contextualized assessments have more predictive validity than non-contextualized personality tests do (Cervone, 2004). Moreover, it also supports the view that the same personality dimension in different scenarios will produce different levels of predictive validity for the same variable (Pathki et al., 2021). This has tremendous significance for future research.

## Limitations and Directions for Future Research

First, the sample of this research was recruited online. Consequently, our sample is skewed to the young and well-educated group. A good measurement tool should have good applicability among different populations. Therefore, future studies should adopt more diverse data collection methods and conclude with more ecological validity by analyzing samples with good generalizability. Second, it is valuable to discuss the relationship and difference between contextualized and non-contextualized personality measurement. However, the direction of discussion in this study is limited to comparing existing studies and does not use different versions of personality scales for comparison. Future research can use various measurement methods to collect data at different time points to conduct a more in-depth study on the stability and differences of personality. Third, from a cross-cultural perspective, cultural factors have an irreplaceable impact on individual personality. Although this study has confirmed that the ORG-B5 has good psychometric properties in the Chinese cultural context, future research can develop other personality scales based on the Chinese cultural context, which may yield different results.

## Data Availability Statement

The original contributions presented in the study are included in the article/Supplementary Material, further inquiries can be directed to the corresponding author.

## Ethics Statement

Ethical review and approval was not required for the study on human participants in accordance with the local legislation and institutional requirements. Written informed consent for participation was not required for this study in accordance with the national legislation and the institutional requirements.

## Author Contributions

All authors have made substantial intellectual contribution to the study, and approved it for publication.

## Funding

This research was funded by Major Research Project of Philosophy and Social Sciences in Colleges and Universities in Henan Province (Grant No.2022-YYZD-20), Soft Science Research Project of Department of Science and Technology of Henan Province (grant no. 202400410094), National Natural Science Foundation of China (grant no. 71772171) and Public Computing Cloud, Renmin University of China.

## Conflict of Interest

The authors declare that the research was conducted in the absence of any commercial or financial relationships that could be construed as a potential conflict of interest.

## Publisher’s Note

All claims expressed in this article are solely those of the authors and do not necessarily represent those of their affiliated organizations, or those of the publisher, the editors and the reviewers. Any product that may be evaluated in this article, or claim that may be made by its manufacturer, is not guaranteed or endorsed by the publisher.
